# Reduced Cardiovascular Capacity and Resting Metabolic Rate in Men with Prostate Cancer Undergoing Androgen Deprivation: A Comprehensive Cross-Sectional Investigation

**DOI:** 10.1155/2015/976235

**Published:** 2015-10-26

**Authors:** Bradley A. Wall, Daniel A. Galvão, Naeem Fatehee, Dennis R. Taaffe, Nigel Spry, David Joseph, Robert U. Newton

**Affiliations:** ^1^School of Psychology and Exercise Science, Murdoch University, Murdoch, WA 6150, Australia; ^2^Exercise Medicine Research Institute, Edith Cowan University, Joondalup, WA 6027, Australia; ^3^School of Medicine, University of Wollongong, Wollongong, NSW 2522, Australia; ^4^Department of Radiation Oncology, Sir Charles Gairdner Hospital, Nedlands, WA 6009, Australia; ^5^Faculty of Medicine, University of Western Australia, Nedlands, WA 6009, Australia; ^6^Centre for Clinical Research, The University of Queensland, Herston, QLD 4006, Australia

## Abstract

*Objectives*. To investigate if androgen deprivation therapy exposure is associated with additional risk factors for cardiovascular disease and metabolic treatment-related toxicities. *Methods*. One hundred and seven men (42–89 years) with prostate cancer undergoing androgen deprivation therapy completed a maximal graded objective exercise test to determine maximal oxygen uptake, assessments for resting metabolic rate, body composition, blood pressure and arterial stiffness, and blood biomarker analysis. A cross-sectional analysis was undertaken to investigate the potential impact of therapy exposure with participants stratified into two groups according to duration of androgen deprivation therapy (<3 months and ≥3 months). *Results*. Maximal oxygen uptake (26.1 ± 6.0 mL/kg/min versus 23.2 ± 5.8 mL/kg/min, *p* = 0.020) and resting metabolic rate (1795 ± 256 kcal/d versus 1647 ± 236 kcal/d, *p* = 0.005) were significantly higher in those with shorter exposure to androgen deprivation. There were no differences between groups for peripheral and central blood pressure, arterial stiffness, or metabolic profile. *Conclusion*. Three months or longer exposure to androgen deprivation therapy was associated with reduced cardiorespiratory capacity and resting metabolic rate, but not in a range of blood biomarkers. These findings suggest that prolonged exposure to androgen deprivation therapy is associated with negative alterations in cardiovascular outcomes. Trial registry is: ACTRN12609000200280.

## 1. Introduction

Androgen deprivation therapy (ADT) is a commonly used treatment for prostate cancer [1]. Although ADT improves prostate cancer survival, a number of studies have reported associations between ADT and treatment-related toxicities, such as increased risk of cardiovascular disease (CVD) and metabolic complications which can compromise survival and quality of life [2–4]. Moreover, several toxicities have been reported to be present soon after initiation of treatment. For example, increased arterial stiffness and adverse body compositional changes were associated with increasing insulin concentrations after 6 months of ADT treatment suggestive of impaired insulin sensitivity [5], and others have reported increases in serum insulin levels after only 3 months of ADT exposure [6]. ADT-related decline in physical function, grip strength, and self-reported physical function has been previously reported by Alibhai et al. [7] when compared with non-ADT controls; ADT users also had worse role physical function, bodily pain, and vitality. Importantly, these reductions in objective and self-reported measures of physical function were apparent within the first 3 months of initiating ADT treatment and persisted for at least 12 months.

Metabolic implications of long-term ADT have been previously explored by Basaria et al. [3], who found men receiving >12 months ADT developed insulin resistance and hyperglycaemia, independent of age and body mass index (BMI). In addition, long-term ADT has been associated with diabetes and metabolic syndrome [8]. Keating et al. [9] were the first to report higher risks of cardiovascular and metabolic complications as well as sudden cardiac death in prostate cancer patients receiving ADT, whilst short-term ADT treatment was significantly associated with greater risks of disease and the elevated risks persisted in men on longer duration therapy [9]. Given the association between cardiorespiratory fitness and CVD mortality [10], the measurement of aerobic capacity can significantly improve the risk classification for CVD mortality [11]. While previous research has predominantly used surrogate measures of cardiorespiratory fitness such as the six-minute walk test (6 MWT) or 400 m walk [7, 12], directly assessing aerobic capacity in ADT-treated men will further our understanding of the cardiovascular risks associated with this form of treatment. In this study, we report for the first time potential differences in relation to patients ADT exposure using objective measures of aerobic capacity, resting metabolic rate, blood pressure, arterial stiffness, and blood biomarkers. We hypothesized that longer-term ADT would lead to compromised aerobic capacity and metabolic parameters hence posing a greater risk for the development and progression of cardiovascular and metabolic diseases.

## 2. Methods

### 2.1. Participants

272 patients with prostate cancer were screened for participation in a 12-month exercise trial [13] from February 2009 to August 2011 in Perth, Western Australia, and Brisbane, Queensland. 109 patients declined participation or were excluded for the following main reasons: declined to participate, too far to travel, unable to fit in with work, unable to obtain general practitioner/physician consent, and bone metastatic disease. In this report, we present the results from baseline assessment of a subgroup of 107 patients who undertook testing including cardiorespiratory capacity by October 2010. This study was approved by the University Human Research Ethics Committee and all participants provided signed informed consent.

All participants underwent assessment for cardiorespiratory capacity, resting metabolic rate, peripheral and central blood pressure and arterial stiffness, and markers of metabolic health.

### 2.2. Cardiorespiratory Capacity

Participants performed a standardised progressive maximal walking test (Bruce Protocol) on a motorized treadmill supervised by a physician. Expired respiratory gases were collected (Parvo Metabolic Measuring System, Sandy, UT, USA) to determine maximal oxygen uptake (the maximal amount of oxygen that can be consumed and utilised, VO_2max_). A plateau in oxygen consumption was used as the criterion for achieving VO_2max_; if no plateau occurred, then a respiratory exchange ratio (RER) of ≥1.1 was used. If the subject achieved no plateau in oxygen consumption or a RER value < 1.1 their data were excluded. This direct assessment of peak oxygen consumption is considered the gold standard outcome of cardiorespiratory fitness or aerobic capacity [14]. The coefficient of variation for repeated maximal exercise tests is approximately 4% [15]. Blood pressure was measured during the last minute of each 3-minute stage via manual auscultatory technique.

### 2.3. Resting Metabolic Rate

Resting metabolic rate (RMR) was measured via respiratory gas analysis over 20 minutes. A 5-minute period that showed an oxygen consumption with a coefficient of variation of <10% was selected for analysis [16]. The coefficient of variation for RMR is <3%.

### 2.4. Resting Blood Pressure and Arterial Stiffness

Brachial blood pressure was recorded at the dominant arm in triplicate via a validated oscillometric device (HEM-705CP, Omron Corporation, Japan) [17]. Applanation tonometry (SPC-301, Millar Instruments, Houston, Texas, USA) was used to measure radial artery pressure waveforms at the right arm. A generalised transfer function was applied to obtain the central pressure waveform at the ascending aorta. Pulse wave analysis was used to determine central blood pressure and indices of arterial stiffness, performed using SphygmoCor version 6.1 software (AtCor Medical, Sydney, Australia). Assessing central blood pressure using this method has been validated against invasive techniques [18]. The augmentation index (AIx) refers to the ratio of augmentation to central pulse pressure, expressed as a percentage, and measures systemic arterial stiffness. Variability has been previously reported as 0.3 mmHg for central systolic pressure and 1.5% for the AIx. Carotid-to-radial pulse wave velocity was measured by collecting arterial pressure waves at both the carotid and radial locations. The reported coefficient of variation for forearm (radial) pulse wave velocity is 2.9% whilst the brachial pulse wave velocity coefficient of variation is 7.7% [19].

### 2.5. Metabolic Syndrome

Patients were classified as having metabolic syndrome if they met three of the following five criteria according to the Adult Panel III Criteria [20]: (1) plasma glucose level more than 110 mg/dL, (2) serum triglyceride levels ≥150 mg/dL, (3) serum high density lipoprotein less than 40 mg/dL, (4) waist circumference greater than 102 cm, and (5) blood pressure ≥135/80 mmHg. Patients on antihypertensive and antilipid medications were also considered positive for the respective criterion.

### 2.6. Other Measures

Venous blood samples (2 × 8.5 mL) were obtained from the antecubital vein with whole blood analysed for haemoglobin A1C (HbA1C, %) whilst the remaining blood was separated and analysed for testosterone, insulin, prostate specific antigen, triglycerides, LDL cholesterol, HDL cholesterol, total cholesterol, glucose, and C-reactive protein. All blood variables were analysed commercially by accredited Australian National Association of Testing Authorities laboratories (Pathwest Laboratory Medicine, WA).

Whole body bone mineral-free lean mass and fat mass, trunk fat, and body fat percentage were assessed by dual energy X-ray absorptiometry (DEXA, Hologic Discovery A, Waltham, MA, USA). In addition, appendicular lean mass was calculated as the sum of upper and lower limb lean mass. The coefficient of variation for body composition measures is <1%.

### 2.7. Statistical Analysis

Sample size calculations for the initial RCT [13] resulted in a requirement for 65 subjects per group at the commencement of the study. For the principal analyses in this report, we had 80% power to detect a significant difference in METS and similarly for VO_2max_ in absolute (L/min) and relative terms (mL/kg/min), and 87% for RMR in kcal/24 hr.

Analyses were performed using the Statistical Package for Social Sciences version 18.0 software (PASW, Chicago, IL, USA). Normality of the data was assessed using the Kolmogorov-Smirnov test. The analyses included standard descriptive statistics, Student's independent *t*-tests, and Pearson's chi-square test for categorical variables. Potential differences between patients on <3 months or ≥3 months on ADT were undertaken based on the previous prospective work by Alibhai and colleagues [7] which showed that even short-term treatment leads to substantial deterioration in physical function. All tests were two-tailed and an alpha level of 0.05 was applied as the criterion for statistical significance. Results are reported as the mean ± standard deviation. 

## 3. Results

### 3.1. Subject Characteristics

Characteristics for all participants were age 68.6 ± 8.8 kg; height 172.4 ± 6.3 cm; and body mass 84.0 ± 13.7 kg. Mean ADT duration was 2.0 ± 0.0 and 7.1 ± 6.2 months for the shorter and longer groups, respectively. There were no significant differences in any subject characteristics (Table 1) or lean and fat mass (Table 2) based on ADT exposure.

### 3.2. Cardiorespiratory Capacity

**Figure 1:**
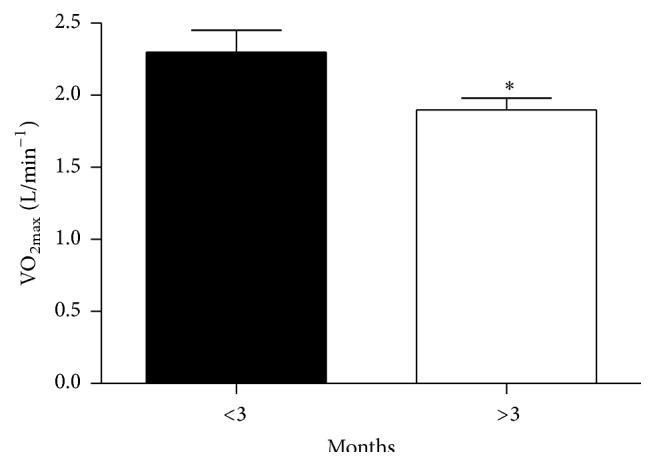
Absolute VO_2max_ values (mean ± SE) for the short- and longer-term androgen deprivation groups. *∗* denotes significant difference versus <3 months.

91 (85%) of the participants were able to achieve the desired criteria for VO_2max_ with no difference between groups in their ability to achieve VO_2max_. Maximal oxygen consumption was significantly higher in the shorter ADT duration group when presented in absolute (L/min^−1^) (*p* = 0.035) (Figure 1) or relative terms (mL/kg/min^−1^) (*p* = 0.020) (Table 3). Corresponding metabolic equivalents were also significantly higher in the shorter duration ADT group (*p* = 0.02). Whilst test duration was not statistically significant, shorter duration group exhibited an additional 54 seconds (*p* = 0.080) of walking endurance.

### 3.3. Resting Metabolic Rate

There was a significant difference observed in RMR with the shorter duration group recording a significantly higher (*p* = 0.005) RMR in absolute terms and relative to body mass RMR (*p* = 0.017) compared to the longer duration group (Table 3). Whilst not statistically significant (*p* = 0.079), RMR relative to lean body mass was 1.3 kcal/lean kg/24 hr higher in those with shorter ADT exposure.

### 3.4. Central Blood Pressure

There were no differences between short and longer ADT exposure in any of the central or peripheral blood pressure variables or central augmentation index (Table 3). Further, there was no difference in pulse wave velocity between groups (Table 3).

### 3.5. Metabolic Profile

No significant differences were observed between short and longer ADT exposure in any of the blood markers analysed (Table 2). Whilst not statistically significant, insulin was 19.6% higher in the shorter duration group (*p* = 0.085).

### 3.6. Metabolic Syndrome Variables

According to National Cholesterol Education Program Adult Treatment Panel III, 20.3% of the acute group and 13.5% of the chronic group were classified as having metabolic syndrome (*p* = 0.337). However, there were no significant differences observed between groups for fasting plasma glucose (*p* = 0.998), serum triglycerides (*p* = 0.874), serum HDL (*p* = 0.815), waist circumference (*p* = 0.994), or hypertension (*p* = 0.093).

## 4. Discussion

We examined the difference between patients on either short- or longer-term ADT across a variety of cardiovascular and metabolic parameters to determine if additional therapy time exposure is associated with accumulating CVD risk factors or metabolic treatment-related toxicities. Those exposed to ADT for a longer period of time had a lower cardiorespiratory capacity and RMR suggesting that longer ADT duration is associated with decline in cardiovascular capacity and aspects of metabolic function.

The maximal aerobic exercise testing protocol used in this study has been shown to be safe and feasible in this population [21]. To our knowledge, this is the first research study to directly measure VO_2max_ in men on ADT to investigate the effect duration of treatment has on cardiorespiratory capacity. Our findings demonstrate that there are differences in cardiorespiratory capacity between men on shorter- and longer-term ADT. The VO_2max_ of the shorter duration ADT group was 21% higher than the longer exposed group, which has important implications when considering that the low levels of cardiorespiratory fitness have been associated with a markedly increased risk of premature death from all causes and in particular CVD in all populations including healthy older men and those with established cardiovascular disease [10]. Conversely, an increase in cardiorespiratory fitness is associated with a reduced risk of CVD [22]. No differences were observed in any of the blood pressure parameters in relation to ADT exposure.

ADT exposure time appears to influence resting energy expenditure with those men on longer duration ADT having a lower RMR. Fat-free mass plays a major role in the variance of RMR amongst individuals [23], with more recent research suggesting that both fat-free mass and fat mass significantly influence RMR [24]. Whilst no significant differences in any body composition values were reported, it is likely that the combined effects of the nonsignificant differences in lean body mass (2 kg) and fat mass (1.5 kg) contributed to this significant reduction in resting energy expenditure in the longer ADT exposure group. A reduced RMR with continuing ADT exposure would contribute to the accumulation of adipose tissue if dietary intake remained unchanged.

When exploring additional CVD risk factors, metabolic syndrome has been widely used as a surrogate marker for CVD. In our study, 20.3% and 13.5% of the shorter and longer ADT exposure groups, respectively, met the criteria for metabolic syndrome. These values are lower than the 55% of ADT-treated prostate cancer patients previously reported by Braga-Basaria et al. [25] but similar to non-ADT-treated prostate cancer patients (22%) and control subjects (20%) reported in the same study [25]. Waist circumference and hypertension appear to be the two most common cardiovascular risk factors present in both groups, demonstrating that these risk factors are present irrespective of the treatment duration.

Whilst not measured in the present study, physical activity levels are known to exert large influences on aerobic capacity and functional performance. Previous research in breast cancer patients has demonstrated that physical activity levels are significantly reduced following cancer diagnosis and during treatment [26, 27] and these reductions in physical activity negatively alter energy balance. We have recently reported that only ~12% of Australian prostate cancer survivors are meeting sufficient exercise levels (150 min of moderate intensity or 75 min of strenuous exercise per week and twice weekly resistance exercise) [28]. It is possible that in the current study physical activity levels were reduced following diagnosis and either they continued to decline as treatment time progressed or the side effects associated with declining physical activity levels did not present until later in treatment which may have contributed to the differences observed in aerobic fitness and RMR.

It was somewhat surprising that we observed no differences in the measured blood biomarkers or central blood pressure in relation to ADT exposure. With regard to the blood biomarkers, it may be that testosterone suppression has such a rapid effect, shown by the increased serum insulin levels previously reported by Dockery et al. [6] after only 3 months of ADT, which leads us to believe that these biomarkers may have stabilised within the first three months.

The strength of this study is that we directly measured VO_2_ during a maximum exercise stress test (gold standard assessment for aerobic capacity) rather than use of surrogate measures such as the 400 meters or 6 MWT as well as assessments of RMR and central blood pressure. A limitation of this study is that patients were only assessed at a single time point during their treatment; hence, we were unable to continually monitor each participant as treatment time progressed. Further, the cross-sectional nature of the study does not permit us to infer causality; however, it provides initial evidence of decline in aerobic capacity measured objectively and it could be clinically meaningful to patients but needs further validation in prospective studies. However, it should be recognised that men in this study were volunteers for an exercise trial and therefore are not representative of all men with prostate cancer undergoing ADT. Lastly, given the exploratory nature of the study, we did not adjust for multiple comparisons [29] and, as a result, cannot discount the possibility that 1-2 of the significant differences may have been a chance finding.

## 5. Conclusion

In conclusion, we found that patients exposed to longer duration ADT had reduced cardiorespiratory capacity and RMR and these could have clinical meaningful implications. The exact mechanisms remain unclear as to why these cardiovascular parameters are further declining as the treatment time progresses and should be determined in future mechanistic studies. Intervention strategies to preserve cardiovascular capacity and RMR such as exercise medicine interventions have significant potential to counteract these forms of decline.

## Figures and Tables

**Table 1:** Subject characteristics for the short- and longer-term androgen deprivation groups.

Variable	<3 months Mean ± SD *n* = 57	≥3 months Mean ± SD *N* = 50	Mean difference (95% CI)	*p* value
Age (years)	67.6 ± 8.9	69.9 ± 9.7	−2.3 (−5.9, 1.3)	0.395
Prostate specific antigen (ng·mL^−1^)	1.2 ± 1.7	1.4 ± 2.6	−0.2 (−1.1, 0.7)	0.659
Gleason Score	7.6 ± 0.8	7.7 ± 1.4	−0.1 (−0.6, 0.6)	0.985
Testosterone (pg·mL^−1^)	1.3 ± 1.5	1.5 ± 3.4	−0.2 (−1.2, 0.8)	0.651
Height (cm)	172.2 ± 6.4	172.7 ± 6.3	−0.5 (−3.2, 2.1)	0.648
Body mass (kg)	85.7 ± 13.6	83.4 ± 14.0	2.3 (−3.1, 7.8)	0.105
Body mass index (kg·m^2^)	28.9 ± 4.3	28.0 ± 3.9	0.9 (−0.7, 2.4)	0.215
Waist circumference (cm)	99.2 ± 12.1	100.3 ± 12.9	−0.9 (−5.5, 3.9)	0.737

**Table 2:** Body composition and blood markers for the short- and longer-term androgen deprivation groups.

Variable	<3 months Mean ± SD *n* = 57	≥3 months Mean ± SD *N* = 50	Mean difference (95% CI)	*p* value
Lean tissue mass (kg)				
Whole body	60.1 ± 7.5	58.0 ± 8.6	2.1 (−1.0, 5.2)	0.184
Appendicular	25.6 ± 3.3	24.5 ± 3.9	1.1 (−0.3, 2.5)	0.112
Fat mass (kg)				
Whole body	23.1 ± 7.3	24.6 ± 8.3	−1.5 (−4.5, 1.4)	0.307
Trunk	12.4 ± 4.5	12.9 ± 5.8	−0.5 (−2.5, 1.5)	0.627
Body fat %	26.4 ± 5.1	28.3 ± 5.2	−1.9 (−4.0, 0.1)	0.053
Blood markers				
HbA1C (%)	6.4 ± 3.7	6.1 ± 1.0	0.3 (−0.8, 1.3)	0.638
Testosterone (pg·mL^−1^)	1.3 ± 1.5	1.5 ± 3.4	−0.2 (−1.2, 0.8)	0.651
Prostate specific antigen (ng·mL^−1^)	1.2 ± 1.7	1.4 ± 2.6	−0.2 (−1.1, 0.7)	0.659
Insulin (mU/L)	11.2 ± 6.7	9.2 ± 4.3	2.0 (−0.3, 4.2)	0.085
Triglycerides (mmol/L)	1.3 ± 0.6	1.4 ± 0.7	−0.1 (−0.3, 0.1)	0.393
LDL cholesterol (mmol/L)	2.8 ± 0.9	2.9 ± 1.0	−0.1 (−0.5, 0.3)	0.575
HDL cholesterol (mmol/L)	1.3 ± 0.4	1.4 ± 0.4	−0.1 (−0.3, 0.1)	0.269
Total cholesterol (mmol/L)	4.7 ± 1.1	4.9 ± 1.0	−0.2 (−0.6, 0.2)	0.287
Glucose (mmol/L)	5.5 ± 1.1	5.9 ± 1.9	−0.4 (−1.0, 0.2)	0.216
C-reactive protein (mg/L)	2.9 ± 3.3	2.5 ± 2.0	0.4 (−0.7, 1.5)	0.469

HbA1C: glycated haemoglobin; LDL: low density lipoprotein; HDL: high density lipoprotein.

**Table 3:** Resting and maximal cardiorespiratory values and hemodynamic and pulse wave analysis parameters for the short- and longer-term androgen deprivation groups.

Variable	<3 months Mean ± SD *n* = 57	≥3 months Mean ± SD *N* = 50	Mean difference (95% CI)	*p* value
VO_2max_ (L/min^−1^)^*∗*^	2.3 ± 1.0	1.9 ± 0.6	0.4 (0.1, 0.7)	0.035
VO_2max_ (mL/kg/min^−1^)^*∗*^	26.1 ± 6.0	23.2 ± 5.8	2.9 (0.5, 5.3)	0.020
VO_2max_ (METS)^*∗*^	7.5 ± 1.7	6.6 ± 1.6	0.9 (0.1, 1.5)	0.020
Test duration (mins)	8.5 ± 2.8	7.6 ± 2.6	0.9 (−0.1, 2.1)	0.080
Resting metabolic rate (kcal/24 hr)^*∗*^	1795 ± 256	1647 ± 236	147 (46, 249)	0.005
Relative total body mass Resting metabolic rate (kcal/kg/24 hr)^*∗*^	21.5 ± 3.0	20.0 ± 2.6	1.5 (0.3, 2.7)	0.017
Relative lean body mass Resting metabolic rate (kcal/lean kg/24 hr)	30.5 ± 3.2	29.2 ± 4.1	1.3 (−0.2, 2.9)	0.079
Peripheral SBP (mmHg)	150.9 ± 19.9	149.0 ± 19.4	1.9 (−5.8, 9.6)	0.624
Peripheral DBP (mmHg)	85.6 ± 12.1	84.5 ± 10.4	1.1 (−3.4, 5.6)	0.626
Peripheral MAP (mmHg)	108.4 ± 15.0	107.2 ± 12.8	1.2 (−4.3, 6.7)	0.669
Central SBP (mmHg)	139.1 ± 21.1	138.7 ± 20.2	0.4 (−7.7, 8.5)	0.922
Central DBP (mmHg)	86.8 ± 12.3	85.5 ± 10.6	1.3 (−3.2, 5.8)	0.571
Central MAP (mmHg)	108.9 ± 16.1	107.2 ± 12.8	1.7 (−4.1, 7.5)	0.557
Peripheral augmentation index (AIx, %)	83.5 ± 13.0	86.5 ± 13.9	−3.0 (−8.3, 2.3)	0.261
Central augmentation pressure (mmHg)	15.4 ± 8.2	16.7 ± 9.6	−1.3 (4.8, 2.1)	0.445
Augmentation load (mmHg)	14.3 ± 5.0	14.6 ± 4.9	−0.3 (−2.3, 1.6)	0.732
Central augmentation index (AIx, %)	140.4 ± 17.8	144.4 ± 19.6	−4.0 (−11.3, 3.3)	0.281
Pulse wave velocity (m/s)	10.0 ± 1.4	10.0 ± 1.8	0.0 (−0.6, 0.7)	0.983

VO_2max_: maximal oxygen uptake; METS: metabolic equivalents; SBP: systolic blood pressure; DBP: diastolic blood pressure; MAP: mean arterial pressure; AIx: augmentation index.

^*∗*^refers to a significant difference between <3 months and ≥3 months.
